# Bed Bugs (*Cimex lectularius*) as Vectors of *Trypanosoma cruzi*

**DOI:** 10.4269/ajtmh.14-0483

**Published:** 2015-02-04

**Authors:** Renzo Salazar, Ricardo Castillo-Neyra, Aaron W. Tustin, Katty Borrini-Mayorí, César Náquira, Michael Z. Levy

**Affiliations:** Chagas Disease Field Laboratory, Universidad Peruana Cayetano Heredia, Arequipa, Peru; Department of Epidemiology, Johns Hopkins Bloomberg School of Public Health, Baltimore, Maryland; Center for Clinical Epidemiology and Biostatistics, University of Pennsylvania School of Medicine, Philadelphia, Pennsylvania

## Abstract

Populations of the common bed bug, *Cimex lectularius*, have recently undergone explosive growth. Bed bugs share many important traits with triatomine insects, but it remains unclear whether these similarities include the ability to transmit *Trypanosoma cruzi*, the etiologic agent of Chagas disease. Here, we show efficient and bidirectional transmission of *T. cruzi* between hosts and bed bugs in a laboratory environment. Most bed bugs that fed on experimentally infected mice acquired the parasite. A majority of previously uninfected mice became infected after a period of cohabitation with exposed bed bugs. *T. cruzi* was also transmitted to mice after the feces of infected bed bugs were applied directly to broken host skin. Quantitative bed bug defecation measures were similar to those of important triatomine vectors. Our findings suggest that the common bed bug may be a competent vector of *T. cruzi* and could pose a risk for vector-borne transmission of Chagas disease.

## Introduction

The resurgence of the common bed bug (*Cimex lectularius*) has created a complex and difficult public health problem. Recent review articles^1^–^3^ as well as national^4^ and international^5^ health agencies have stressed the dermatologic and allergenic consequences of bed bug bites. These same sources note the lack of firm evidence that bed bugs are capable of transmitting human pathogens. As a result, most health authorities, while acknowledging the massive economic impact caused by the insects, treat bed bugs along the same lines as cockroaches: a nuisance and an environmental health issue.^6^ Nonetheless, the hematophagous (blood-feeding) behavior of these insects coupled with their recent and intense proliferation in human habitats present an unambiguous potential for the transmission of infectious diseases.

Bed bugs and triatomine bugs, such as *Triatoma infestans*, share many striking similarities. Both insects hide in household cracks and crevices waiting for nightfall and the opportunity to feed on sleeping hosts. They are from the same order of insects (the *Hemiptera* or true bugs), and both are exclusively hematophagous, although the blood-sucking lifestyle of each insect evolved independently.^7^ It is possible that this convergent evolution includes the trait for which triatomines are infamous: the transmission of *Trypanosoma cruzi*, the etiologic agent of Chagas disease. We performed experiments to evaluate if and how easily *T. cruzi* can be transferred from a mammalian host to bed bugs as well as evaluate if and how easily the parasite can be transferred from infected bed bugs back to a mammalian host. In addition, we recorded movies (Supplemental Videos 1 and 2) of bed bug feeding and defecation patterns to quantify the potential of stercorarian (feces-mediated) transmission of *T. cruzi*.

## Methods

### Bed bugs.

The bed bugs used in this study were members of a colony of *C. lectularius* (Arequipa strain CL-1) raised since 2011 under controlled laboratory conditions (ambient temperature of 25–30°C, relative humidity of 50–70%, and 12-hour photoperiod). Before conducting the experiments, we examined the feces of a random sample of 40 adult bed bugs (20 males and 20 females) for the presence of *T. cruzi*; all were clear of the parasite.

### Extraction and preparation of bed bug feces.

Several of our experiments required the extraction of bed bug feces. To obtain bed bug feces, we adapted a standard technique used to analyze triatomine feces for the presence of *T. cruzi*. We extracted feces from each bed bug by applying pressure to the abdomen with tweezers. We then diluted the feces in 10 μL normal saline.

### Experiment 1: Transmission of *T. cruzi* from mice to bed bugs.

We infected 10 female 1-month-old BALB/c mice (*Mus musculus*) with *T. cruzi* (Arequipa strain TC-35) by intraperitoneal injection with an inoculum size of 10^3^ parasites in 100 μL. Beginning 3 days post-inoculation and continuing every 3 days for 1 month, we evaluated parasitemia by the microhematocrit concentration method.^8^ We drew blood from the tail of each mouse into two heparinized microhematocrit tubes, which we then spun at 7,000 rpm for 2 minutes. We severed the tubes between the buffy coat and the erythrocyte layer and poured the buffy coat and plasma onto microscope slides. Two observers counted parasites by examining 100 microscopic fields at 400× magnification. The number of parasites was expressed in terms of parasites per milliliter of blood.

Every 3 days, we also allowed 20 previously unfed male *C. lectularius*, ranging in age from 7 to 10 days, to feed on each of 10 infected mice. Twenty-one days after feeding, we extracted the feces of these insects as described above. We then examined the diluted feces microscopically at 400× magnification for the presence of *T. cruzi* epimastigotes and trypomastigotes. We recorded the total number of insects infected by each stage of the parasite.

### Experiment 2: Transmission of *T. cruzi* from bed bugs to mice.

To create a population of infected *C. lectularius*, we inoculated four 1-month-old mice with *T. cruzi* by the intraperitoneal route using the strain and methods described in experiment 1. To intensify the degree of parasitemia, we used a larger inoculum size (2 × 10^3^ parasites/100 μL). Two weeks later, after parasitemia in the mice was confirmed, we allowed 75 adult male bed bugs to feed for 15–20 minutes on each mouse (for a total of 300 insects). The same insects were fed again 1 week later to increase the probability of their infection. Two weeks after the second feeding, we examined the feces of a random sample of 10 of 300 exposed insects to confirm that they had acquired the parasite. We observed *T. cruzi* in the feces of all 10 insects.

We created 12 habitats in a small (25 × 20 × 22 cm) aquaria; into each habitat, we placed one 2-month-old uninfected mouse and 20 of the bed bugs that had fed on infected mice. We allowed the mice and bed bugs to cohabit for 30 days. Every 6 days, we tested each mouse for *T. cruzi* parasitemia by the microhematocrit method described above. We also performed xenodiagnosis^9^ every 6 days by allowing 20 uninfected male *C. lectularius* to feed on each mouse; we examined the feces of these bed bugs for parasites 21 days later.

### Experiment 3: Transcutaneous transmission of *T. cruzi* from bed bugs to mice.

During experiment 2, several mice killed and ingested the bed bugs in their aquaria, and mice likely became infected orally. We, therefore, designed an additional experiment to evaluate transcutaneous transmission through contaminated bed bug feces using a protocol described previously.^10^ Briefly, we shaved portions of the backs of 10 2-month-old uninfected mice using an electric razor. The next day, we caused microtrauma to the shaved areas of five of the mice by puncturing the skin with a tuberculin needle. Microtrauma to the other five mice was produced by exposing each mouse to 10 uninfected bed bugs that were allowed to feed on the shaved areas for 20 minutes. We then placed 40 μL diluted feces from infected *C. lectularius* onto the shaved zone of each mouse (in a representative sample of diluted feces, the *T. cruzi* concentration was 280 trypomastigotes per 40 μL). After 10 minutes, we cleaned the areas with sterile saline solution. We analyzed the mice for *T. cruzi* every 6 days for 30 days through the microhematocrit method described above.

### Experiment 4: Observation of bed bug defecation patterns.

We used an artificial feeding system described previously^11^ consisting of a stretched Parafilm (Bemis NA, Neenah, WI) membrane across a glass feeding apparatus. Human blood (obtained from the Division of Transfusion Medicine at the University of Pennsylvania) was warmed to 37°C and placed on top of the Parafilm. Twenty adult bed bugs were placed into the jar, where they were able to take blood meals through the membrane. A sheet of paper lined the walls of the container to provide refuge into which the insects could retreat. The experiment was repeated three times, with each replication lasting between 2 and 3 hours. We used a video camera to record the insects' behavior. We then analyzed the movies to determine the time delay from the end of feeding to defecation (Supplemental Videos 1 and 2).

### Ethics statement.

All animal experiments were conducted at the Chagas Disease Field Station of the Universidad Peruana Cayetano Heredia in Arequipa, Peru. The protocol (identification number 61410) was approved by the Institutional Animal Care and Use Committee of the Universidad Peruana Cayetano Heredia. The protocol adhered to Peruvian and international guidelines, including the Código Deontológico of the Colegio Médico Veterinario del Perú.

## Results

### Experiment 1.

**Figure 1.**
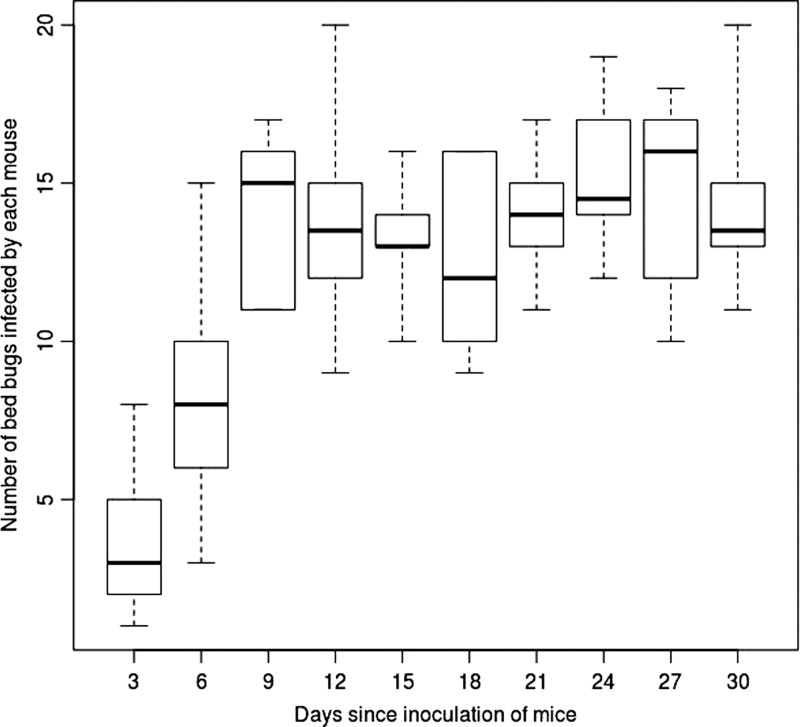
Transmission of *T. cruzi* from mice to bed bugs. Box plot of the number of bed bugs that acquired *T. cruzi* (of 20 total insects exposed to each mouse at each time point) after feeding on infected mice. Whiskers represent minimum and maximum values.

**Figure 2.**
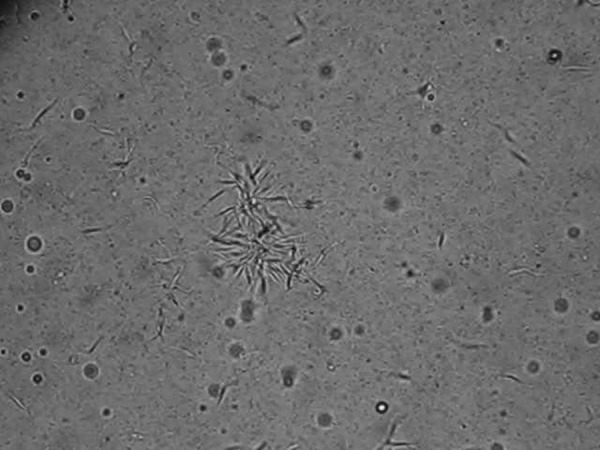
Aggregation of *T. cruzi* in feces of an infected *C. lectularius*.

The majority of bed bugs acquired *T. cruzi* after feeding on infected mice (Figure 1

). Transmission occurred as early as 3 days post-inoculation and persisted throughout 30 days of the experiment. In the intestinal contents of infected *C. lectularius*, we observed both the infective trypomastigote and replicative epimastigote forms of the parasite in extremely high numbers (Figure 2

and Supplemental Videos 1 and 2). Trypomastigotes were present in the intestinal contents of 40% of infected bed bugs.

### Experiment 2.

We first observed a small number of circulating parasites in 2 of 12 exposed mice after 12 days of cohabitation, and eventually, 58% (7 of 12) of mice exhibited parasitemia (Table 1). Parasite counts rose quickly in the infected mice, mirroring concentrations in mice infected by intraperitoneal injection. Xenodiagnosis was slightly more sensitive, detecting *T. cruzi* in 75% (9 of 12) of mice. Two mice that were positive by xenodiagnosis had undetectable parasite counts in peripheral blood.

### Experiment 3.

We found *T. cruzi* in the blood of 40% (4 of 10) of mice with broken skin that was exposed to the feces of infected bed bugs. Parasitemia was present in 60% (three of five) of mice with skin that was punctured by tuberculin needles and 20% (one of five) of mice with microtrauma that was caused by bed bug feeding. Circulating parasites were first detected 12 days after exposure. This result shows the viability of trypomastigotes seen in the intestinal contents of infected bed bugs.

### Experiment 4.

Twelve of sixty (20%) bed bugs fed during the observation period. Of these bed bugs, 10 insects (83%) defecated on the Parafilm membrane near the feeding site, and 1 insect (8%) defecated on the paper refuge. In all cases in which defecation occurred on the membrane, the feces adhered to the membrane rather than falling to the bottom of the container, suggesting an extremely high probability of contact between bed bug feces and human skin during the course of the insects' feeding. We computed the post-feeding defecation time for 10 insects; the insect that defecated on the paper refuge did so out of the field of view of the video camera and was not included in the following analysis. The median time between the end of feeding and the first defecation for these 10 insects was 5.1 minutes (range = 0.3–16.0 min); 1 of 10 insects defecated two times within 10 minutes of feeding. We calculated the defecation index, which for triatomine insects, is defined as the fraction of insects defecating within 10 minutes multiplied by the average number of defecations in 10 minutes.^12^ The defecation index for adult bed bugs was 0.74.

## Discussion

In this study, we show that the common bed bug seems to be a competent vector of *T. cruzi*. Bed bugs efficiently acquired *T. cruzi* on feeding on infected mice. Bed bugs then transmitted the parasite back to susceptible hosts both during cohabitation and through contaminated feces placed on broken host skin by researchers. Our quantitative measurements of *C. lectularius* defecation patterns confirm that bed bugs have a high potential for feces-mediated transmission.

Bed bugs are capable of transmitting *T. cruzi*; whether they will become epidemiologically important vectors of the parasite remains unclear. The key parameters that determine whether a vector-borne pathogen can be expected to spread through a population are codified in mathematical models, such as those proposed by Ross^13^ and Macdonald.^14^ These parameters include the numerical ratio of vectors to hosts, the rate of contact between vectors and hosts, and the probability of infection transmission with each contact.^15^ Notably, the first two of these parameters are higher for bed bugs than triatomine bugs; bed bugs generally reach greater densities in infested households than triatomines^16^,^17^ and feed about two times as often as *T. infestans*.^5^,^18^ The third parameter, the probability of *T. cruzi* transmission per contact, is inversely related to the elapsed time between feeding and defecation^19^ and can be estimated by a proposed defecation index,^12^ with a value that is larger in species with a higher potential for transmission. In our study, adult bed bugs defecated a mean of 6.0 minutes (median = 5.1 minutes) after feeding. This mean time delay can be compared with the mean time delays of adults of three important Latin American triatomine vectors: *T. infestans*, 3.0 minutes; *Rhodnius prolixus*, 8.6 minutes; and *T. dimidiata*, 13.9 minutes.^12^ Adult bed bugs have a defecation index of 0.74, which is intermediate among those of adult *T. dimidiata* (0.55), *T. infestans* (0.95), and *R. prolixus* (1.0).^12^ Based on these parameters, compared with the two most important vectors of Chagas disease, adult bed bugs have a transmission potential similar to that of *R. prolixus* (bed bugs defecate faster but have a lower defecation index) and somewhat less than that of *T. infestans*.

The transmission of *T. cruzi* by bed bugs has been suspected for many years. In 1912, just 3 years after Carlos Chagas described the transmission of *T. cruzi* by triatomines, Brumpt^20^ claimed to have infected almost all of 100 bed bugs exposed to an infectious mouse and subsequently, two mice through exposure to bed bugs. Several decades later, an Argentine group replicated the experimental transmission of *T. cruzi* between mice and bed bugs.^21^,^22^ These reports, written in French and Spanish, respectively, have been overlooked during the recent re-emergence of bed bugs. Critically, these previous works overlooked a key point: mice actively hunt and eat bed bugs, and the transmission that they observed was almost certainly oral.^21^,^22^ Our work improves on these earlier studies by showing that *T. cruzi* in bed bug feces can infect mice through contact with broken skin and that bed bugs often defecate shortly after feeding.

Wild bed bugs in Argentina may also have been found to harbor *T. cruzi*. Jörg^22^ relates that, in 1938, Salvador Mazza isolated the parasite from 4% of bed bugs and 40% of triatomines captured in the city of Jujuy. Such reports must be interpreted cautiously, given recent confusion over *T. cruzi* in wild-caught Mexican “bed bugs” that were, in fact, triatomines.^23^ However, in its original Spanish, the article by Jörg^22^ clearly distinguishes between bed bugs (“chinches”) and triatomines (*T. infestans* or “vinchucas”).^22^ Still, the report is only secondhand^22^; systematic analysis of bed bug populations in areas of *T. cruzi* endemicity will be needed to determine if they do commonly carry the parasite.

It is tempting to believe that bed bugs cannot be epidemiologically relevant vectors of *T. cruzi*, because if they were, then their role in the proliferation of Chagas disease would have been obvious long ago. However, such an argument is tenuous. Pathogen emergence is a stochastic process^24^; even if the conditions to support transmission are present, a disease agent may not appear by simple chance. The conditions for the emergence of *T. cruzi* through bed bug populations are currently and may become particularly conducive to the emergence of *T. cruzi*. Bed bug populations in the United States have increased substantially in the past 15 years.^5^,^25^ Meanwhile, recent estimates suggest that at least 80,000^26^ and possibly, as many as 300,000^27^ residents of the United States currently harbor *T. cruzi* infection. In the United States, the prospect of *T. cruzi* transmission by bed bugs is, therefore, at a historically high level.

Equally troubling is the recent appearance of new populations of bed bugs in areas of *T. cruzi* endemicity in Latin America. This study was motivated in part by reports, which we have confirmed, of emerging populations of *C. lectularius* in the city of Arequipa, Peru, where Chagas disease remains an urban problem.^16^,^28^,^29^ Although common and tropical (*C. hemipterus*) bed bugs have inhabited certain areas of Latin America endemic for Chagas disease, there has been little effort to distinguish transmission of *T. cruzi* caused by bed bugs from that caused by triatomines, perhaps because previously, both insects could be eliminated through household application of pyrethroid insecticides. The bed bug populations that have resurged in recent years are highly resistant to pyrethroids^30^ and other classes of insecticides,^31^ and the cost of eliminating them from houses is an order of magnitude greater than the cost of controlling triatomines.^32^,^33^ If these resurgent bed bugs become prevalent in areas of high Chagas disease burden, they may pose a serious challenge to the elimination of vector-borne transmission of Chagas disease from the southern cone of South America and other regions.

## Supplementary Material

Supplemental Data.

## Figures and Tables

**Table 1:** Transmission of *T. cruzi* from bed bugs to mice

Days since the start of cohabitation	Number (%) of 12 sentinel mice with *T. cruzi* observed in microhematocrits	Parasitemia in sentinel mice: number of parasites per 1 mL blood median (first, third quartiles)
6	0 (0)	0 (0, 0)
12	2 (17)	0 (0, 0)
18	6 (50)	7 (0, 1.1 × 10^3^)
24	7 (58)	2.6 × 10^4^ (0, 3.6 × 10^5^)
30	7 (58)	1.4 × 10^6^ (0, 1.9 × 10^6^)

Twelve aquaria, each with 20 bed bugs previously exposed to *T. cruzi* and a single uninfected mouse, were observed over the course of 1 month.
