# Supplementary material for: Bed Bugs (Cimex lectularius) as Vectors of Trypanosoma cruzi
Source: Am J Trop Med Hyg. 2015 Feb 4;92(2):331–5. doi: 10.4269/ajtmh.14-0483 (PMC4347337; doi:10.4269/ajtmh.14-0483)
Supplement: Supplementary file 1 [file SD3.pdf]

DISCLAIMER: THIS TRANSLATION WAS PROVIDED BY THE AUTHORS AND THE JOURNAL CANNOT GUARANTEE ITS ACCURACY

Chinches de Cama (*Cimex lectularius*) como Vectores de *Trypanosoma cruzi*

Renzo Salazar, Ricardo Castillo-Neyra, Aaron W. Tustin, Katty Borrini-Mayorí, César Náquira, and Michael Z. Levy*

Chagas Disease Field Laboratory, Universidad Peruana Cayetano Heredia, Arequipa, Peru; Department of Epidemiology, Johns Hopkins Bloomberg School of Public Health, Baltimore, Maryland; Center for Clinical Epidemiology and Biostatistics, University of Pennsylvania School of Medicine, Philadelphia, Pennsylvania

* Autor corresponsal: Dr. Michael Z. Levy mzlevy@mail.med.upenn.edu

Resumen. Las poblaciones de chinche de cama común, *Cimex lectularius*, han experimentado últimamente un crecimiento explosivo. Los chinches de cama comparten muchas características importantes con los insectos triatominos, pero aún permanece incierto si estas similitudes incluyen la capacidad de transmitir *Trypanosoma cruzi*, el agente etiológico de la enfermedad de Chagas. Aquí, nosotros mostramos una transmisión eficiente y bidireccional de *T. cruzi* entre hospederos y chinches de cama en un ambiente de laboratorio. La mayor parte de los chinches de cama que se alimentaron de ratones infectados experimentalmente adquirieron el parásito. La mayoría de ratones sin infección previa llegaron a infectarse después de un periodo de convivencia con chinches de cama infectados. *T. cruzi* también fue transmitido a ratones después de que se aplicaron heces de chinches de cama infectados directamente sobre la piel herida. La cantidad de defecaciones de chinches de cama fueron similares a aquellas de vectores triatominos importantes. Nuestros hallazgos sugieren que el chinche de cama común puede ser un vector competente de *T. cruzi* y podría suponer un riesgo para la transmisión vectorial de la enfermedad de Chagas.

INTRODUCCIÓN

El resurgimiento del chinche de cama común (*Cimex lectularius*) ha creado un complejo y difícil problema de salud pública. Recientes artículos de revisión[1-3] así como agencias nacionales[4] e internacionales[5] de salud han puesto énfasis en las consecuencias dermatológicas y alergénicas de las picaduras de los chinches de cama. Estas mismas fuentes señalan la falta de evidencia firme de que los chinches de cama sean capaces de transmitir patógenos humanos. Como resultado, la mayoría de autoridades de salud, aun cuando reconocen el masivo impacto económico causado por los insectos, consideran a los chinches de cama bajo las mismas líneas que las cucarachas: una molestia y un problema de salud

ambiental.[6] Sin embargo, el comportamiento hematófago (alimentación de sangre) de estos insectos unido a su reciente e intensa proliferación en hábitats humanos presenta un potencial inequívoco para la transmisión de enfermedades infecciosas.

Los chinches de cama y los insectos triatominos, como *Triatoma infestans*, comparten muchas notables similitudes. Ambos insectos se ocultan en las grietas y ranuras esperando el anochecer y la oportunidad para alimentarse de hospederos dormidos. Ambos son del mismo orden de insectos (*Hemíptera* o chinches verdaderos), y ambos son exclusivamente hematófagos, aunque el estilo de vida hematófago de cada insecto evolucionó independientemente.[7] Es posible que esta evolución convergente incluya la característica por la cual los triatominos son infames: la transmisión de *Trypanosoma cruzi*, el agente etiológico de la enfermedad de Chagas. Nosotros realizamos experimentos para evaluar si *T. cruzi* puede ser transmitido de un hospedero mamífero a chinches de cama y que tan fácilmente puede ser transmitido, así como evaluar si el parásito puede ser transmitido a partir de un chinche de cama infectado a un hospedero mamífero y que tan fácilmente puede ser transmitido. Adicionalmente, grabamos videos (videos suplementarios 1 y 2) de patrones de alimentación y defecación de chinches de cama para cuantificar el potencial de transmisión estercoraria (mediante heces) de *T. cruzi*.

MÉTODOS

Chinches de cama. Los chinches de cama usados en este estudio fueron miembros de una colonia de *C. lectularius* (cepa Arequipa CL-1) criada desde el 2011 bajo condiciones controladas de laboratorio (temperatura ambiental de 25-30°C, humedad relativa de 50-70%, y 12 horas de fotoperiodo). Antes de realizar los experimentos, examinamos las heces de una muestra aleatoria de 40 chinches de cama adultos (20 machos y 20 hembras) para determinar la presencia de *T. cruzi*; todos estuvieron libres del parásito.

Extracción y preparación de heces de chinches de cama. Varios de nuestros experimentos requirieron la extracción de heces de chinches de cama. Para obtener heces de chinches de cama, adaptamos una técnica estándar usada para analizar heces de triatominos para determinar la presencia de *T. cruzi*. Extrajimos las heces de cada chinche de cama aplicando presión al abdomen con pinzas. Luego diluimos las heces en 10 µl de suero fisiológico.

Experimento 1: Transmisión de *T. cruzi* de ratones a chinches. Infectamos 10 ratones (*Mus musculus*) hembras BALB/c de 1 mes de edad con *T. cruzi* (cepa Arequipa TC-35) por inyección intraperitoneal con un inóculo de 10^3 parásitos en 100 µl. Comenzando a los 3 días post-inoculación y continuando cada 3 días por 1 mes, evaluamos la parasitemia por el método de concentración para microhematocrito.[8] Colectamos la sangre de la cola de cada ratón en dos tubos heparinizados de microhematocrito, los cuales luego fueron centrifugados a 7,000 rpm por 2 minutos. Cortamos los tubos entre la capa leucocitaria y la capa de eritrocitos y vertimos la capa leucocitaria y el plasma en láminas para microscopio. Dos observadores contaron los parásitos examinando 100 campos bajo el microscopio a 400X de

aumento. El número de parásitos fue expresado en términos de parásitos por mililitro de sangre.

Cada 3 días, también dejamos que 20 machos de *C. lectularius*, con un rango de edad de 7 a 10 días, se alimenten de cada uno de los ratones. Veintiún días después de la alimentación, extrajimos las heces de estos insectos como se describió arriba. Luego examinamos las heces diluidas al microscopio a 400X de aumento para determinar la presencia de epimastigotes y tripomastigotes de *T. cruzi*. Se registró el número total de insectos infectados por cada estado del parásito.

Experimento 2: Transmisión de *T. cruzi* de chinches de cama a ratones. Para crear una población de *C. lectularius* infectada, inoculamos por vía intraperitoneal 4 ratones de 1 mes de edad con *T. cruzi*, usando la cepa y el método descrito en el experimento 1. Para intensificar el grado de parasitemia, usamos un mayor inóculo (2×10^3 parásitos en 100 µl). Dos semanas más tarde, después de que la parasitemia en los ratones fue confirmada, dejamos que 75 machos adultos de chinches de cama se alimentaran por 15 - 20 minutos de cada ratón (para un total de 300 insectos). Los mismos insectos fueros alimentados una vez más 1 semana después para incrementar la probabilidad de su infección. Dos semanas después de la segunda infección, examinamos las heces de una muestra aleatoria de 10 de los 300 insectos expuestos para confirmar que habían adquirido el parásito. Se observó *T. cruzi* en las heces de los 10 insectos.

Creamos 12 hábitats en pequeños acuarios (25 x 20 x 22 cm); en cada hábitat colocamos un ratón sin infección y 20 de los chinches de cama que se habían alimentado de los ratones infectados. Dejamos que los ratones y los chinches de cama convivieran por 30 días. Cada 6 días, evaluamos cada ratón para determinar la parasitemia por *T. cruzi* mediante el método de microhematocrito descrito arriba. También aplicamos xenodiagnósticos[9] cada 6 días permitiendo que 20 machos sin infección de *C. lectularius* se alimenten de cada ratón; 21 días después se examinaron las heces de estos chinches de cama para determinar la presencia de parásitos.

Experimento 3: Transmisión transcutánea de *T. cruzi* de chinches de cama a ratones. Durante el experimento 2, varios ratones mataron e ingirieron los chinches de cama en sus acuarios, y los ratones probablemente llegaron a infectarse por vía oral. Por lo tanto, diseñamos un experimento adicional para evaluar la transmisión transcutanea por medio de heces contaminadas de chinches de cama usando un protocolo descrito previamente.[10] Brevemente, se afeitó una porción de la espalda de 10 ratones sin infección de 2 meses de edad usando una rasuradora eléctrica. Al día siguiente, causamos microtraumas al área afeitada de cinco de los ratones por punción de la piel con una aguja de tuberculina. El microtrauma de los otros cinco ratones fue producido por exposición de cada ratón a 10 chinches de cama sin infección que fueron alimentados en el área afeitada por 20 minutos. Luego colocamos en la zona afeitada de cada ratón 40 µl de heces diluidas de *C. lectularius* infectados (en una muestra representativa de heces diluidas, la concentración de *T. cruzi* fue de 280 trypomastigotes en 40 µl). Después de 10 minutos limpiamos el área con suero

fisiológico estéril. Cada 6 días por un periodo de 30 días analizamos a los ratones para determinar la presencia de *T. cruzi* a través del método de microhematocrito descrito arriba.

Experimento 4: Observación de patrones de defecación. Usamos un sistema de alimentación artificial descrito previamente[11] que consiste de una membrana de Parafilm estirada alrededor de un aparato alimentador de vidrio. Sangre humana (obtenida de la División de Medicina de Transfusión de la Universidad de Pensilvania) fue calentada a 37°C y colocada sobre el Parafilm. Veinte chinches de cama adultos fueron colocados en el frasco, donde podían tomar sangre a través de la membrana. Una hoja de papel corrugado cubría las paredes del recipiente para proveer refugio en los cuales los insectos pudieran esconderse. El experimento fue repetido 3 veces, cada repetición duro entre 2 y 3 horas. Se usó una cámara de video para grabar el comportamiento del insecto. Luego analizamos las películas para determinar el tiempo desde el final de la alimentación hasta la defecación. (videos suplementarios 1 y 2).

Consideraciones éticas. Todos los experimentos animales fueron realizados en la Estación de Campo de la Enfermedad de Chagas de la Universidad Peruana Cayetano Heredia en Arequipa, Perú. El protocolo (número de identificación 61410) fue aprobado por el Comité Institucional para el uso y cuidado de animales de la Universidad Peruana Cayetano Heredia. El protocolo se adhirió a las directrices Peruanas e Internacionales, incluyendo el Código Deodontológico del Colegio Médico Veterinario del Perú.

RESULTADOS

Experimento 1. La mayoría de chinches de cama adquirieron *T. cruzi* después de alimentarse en ratones infectados (figura 1). La transmisión ocurrió desde los 3 días post inoculación y persistió a lo largo de los 30 días del experimento. En el contenido intestinal de *C. lectularius* infectados, observamos ambas formas del parásito, el tripomastigote infectivo, y el epimastigote replicativo, en números extremadamente elevados (figura 2 y videos suplementarios 1 y 2). Los tripomastigotes estuvieron presentes en el contenido intestinal del 40% de los chinches de cama infectados).

Experimento 2. Primero observamos un pequeño número de parásitos circulantes en 2 de 12 ratones expuestos después de 12 días de convivencia, y eventualmente, el 58% (7 de 12) de los ratones mostraron parasitemia (tabla 1). Los conteos de parásitos aumentaron rápidamente en los ratones infectados, replicando concentraciones en ratones infectados por inyección intraperitoneal. El xenodiagnóstico fue ligeramente más sensitivo, detectando *T. cruzi* en el 75% (9 de 12) de los ratones. Dos ratones que fueron positivos por xenodiagnóstico tuvieron conteos de parásitos indetectable en sangre periférica.

Experimento 3. Encontramos *T. cruzi* en la sangre del 40% (4 de 10) de los ratones con piel herida que fueron expuestos a las heces de chinches de cama infectados. La parasitemia estuvo presente en el 60% (tres de cinco) de los ratones con la piel punzada por agujas de tuberculina y el 20 % (uno de cinco) de los ratones con microtrauma provocado por la

alimentación de los chinches de cama. Los parásitos circulantes fueron detectados por primera vez a los 12 días después de la exposición. Estos resultados muestran la viabilidad de los tripomastigotes observados en el contenido intestinal de los chinches de cama infectados.

Experimento 4. Doce de sesenta (20%) chinches de cama se alimentaron durante el periodo de observación. De estos chinches de cama, 10 insectos (83%) defecaron en la membrana de Parafilm cerca del sitio de alimentación, y 1 insecto (8%) defecó en el papel del refugio. En todos los casos en los cuales la defecación ocurrió en la membrana, las heces se adhirieron a la membrana en vez de que cayeran al fondo del recipiente, sugiriendo una probabilidad extremadamente alta de contacto entre las heces del chinche de cama y la piel humana durante el curso de la alimentación del insecto. Calculamos el tiempo de defecación post-alimentación para 10 insectos; el insecto que defecó en el papel del refugio lo hizo fuera del campo de visión de la cámara de video y no fue incluido en el análisis. El tiempo promedio entre el final de la alimentación y la primera defecación para estos 10 insectos fue de 5.1 minutos (rango= 0.3 - 16 min); 1 de los 10 insectos defecó 2 veces en los 10 minutos de alimentación. Calculamos el índice de defecación, el cual para insectos triatominos, es definido como la fracción de insectos defecando en el lapso de 10 minutos multiplicado por el número promedio de defecaciones en 10 minutos.[12] El índice de defecación para chinches de cama adultos fue 0.74.

DISCUSIÓN

En este estudio, mostramos que el chinche de cama común parece ser un vector competente de *T. cruzi*. Los chinches de cama adquirieron eficientemente *T. cruzi* al alimentarse de ratones infectados. Luego los chinches de cama transmitieron el parásito de regreso a hospederos susceptibles durante la convivencia y a través de heces contaminadas colocadas sobre la piel herida del hospedero provocada por los investigadores. Nuestras mediciones cuantitativas de los patrones de defecación de *C. lectularius* confirman que los chinches de cama tienen un elevado potencial para la transmisión mediante heces.

Los chinches de cama son capaces de transmitir *T. cruzi*; que ellos lleguen a ser epidemiológicamente importantes vectores del parásito permanece incierto. Los parámetros clave que determinan si un patógeno de transmisión vectorial puede dispersarse a través de una población están codificados en modelos matemáticos, así como aquellos propuestos por Ross[13] y Macdonald.[14] Estos parámetros incluyen la razón numérica de vectores a hospederos, la tasa de contacto entre vectores y hospederos, y la probabilidad de transmisión de la infección con cada contacto. Notablemente, los primeros dos de estos parámetros son más elevados para chinches de cama que para insectos triatominos; los chinches de cama generalmente alcanzan mayores densidades en viviendas infestadas que los triatominos[16,17] y se alimentan alrededor de dos veces más a menudo que *T. infestans*.[5,18] El tercer parámetro, la probabilidad de transmisión de *T. cruzi* por contacto, está inversamente relacionada al periodo de tiempo entre la alimentación y la defecacion[19] y puede ser estimada por un índice de defecación propuesto,[12] con un valor que es mayor en especies con un alto potencial para la

transmisión. En nuestro estudio, los chinches de cama adultos defecaron en promedio 6.0 minutos (mediana = 5.1 minutos) después de la alimentación. Este tiempo promedio puede ser comparado con el tiempo promedio de adultos de tres importantes vectores triatominos en América Latina: *T. infestans*, 3.0 minutos; *Rhodnius prolixus*, 8.6 minutos y *T. dimidiata*, 13.9 minutos.[12] Los chinches de cama adultos tienen un índice de defecación de 0.74, el cual es intermedio entre los adultos de *T. dimidiata* (0.55), *T. infestans* (0.95) y R. prolixus (1.0).[12] Basándonos en estos parámetros, comparado con los dos vectores más importantes de la enfermedad de Chagas, los chinches de cama adultos tienen un potencial de transmisión similar al de *R. prolixus* (los chinches de cama defecaron más rápido pero tienen un menor índice de defecación) y algo menos que el de *T. infestans*.

La transmisión de *T. cruzi* por chinches de cama ha sido sospechada por muchos años. En 1912, solo 3 años después de que Carlos Chagas describiera la transmisión de *T. cruzi* por triatominos, Brumpt[20] declaró haber infectado casi todos los 100 chinches de cama expuestos a un ratón infectado y luego, de haber infectado dos ratones a través de la exposición a chinches de cama. Varias décadas después, un grupo argentino replicó la transmisión experimental de *T. cruzi* entre ratones y chinches de cama.[21, 22] Estos reportes, escritos en Francés y Español, respectivamente, han sido pasados por alto durante la reciente re-emergencia de los chinches de cama. Críticamente, estos trabajos previos pasaron por alto un punto clave: los ratones cazan activamente y comen chinches de cama, y la transmisión que ellos observaron fue casi con seguridad oral.[21, 22] Nuestro trabajo mejora con respecto a estos estudios tempranos al mostrar que *T. cruzi* en heces de chinches de cama pueden infectar ratones a través del contacto con piel dañada y que los chinches de cama a menudo defecan poco después de la alimentación.

Chinches de cama silvestres en Argentina también pudieron haber sido encontrados albergando *T. cruzi*. Jörg[22] relata que, en 1938, Salvador Mazza aisló el parasito del 4% de los chinches de cama y el 40% de triatominos capturados en la ciudad de Jujuy. Tales reportes deben ser interpretados cuidadosamente, debido a la reciente confusión sobre *T. cruzi* en "chinches de cama" silvestres capturados en México, que de hecho, fueron triatominos.[23] Sin embargo, en su español original, el artículo de Jörg[22] claramente distingue entre chinches de cama ("chinches") y triatominos (*T. infestans* o "vinchucas").[22] Aun así , el reporte es solamente de segunda mano; análisis sistemáticos de las poblaciones de chinches de cama en áreas endémicas de *T. cruzi* serán necesario para determinar si ellos comúnmente portan el parásito.

Es tentador creer que los chinches de cama no pueden ser vectores epidemiológicamente relevantes de *T. cruzi*, debido a que si lo fueran, entonces su rol en la proliferación de la enfermedad de Chagas hubiera sido obvio hace mucho. Sin embargo, tal argumento es tenue. La emergencia de patógenos es un proceso estocástico[24]; incluso si las condiciones para apoyar la transmisión están presentes, un agente patógeno no puede aparecer por simple casualidad. Las condiciones para la emergencia de *T. cruzi* a través de poblaciones de chinches de cama son actualmente y pueden llegar a ser particularmente propicias para la emergencia de *T. cruzi*. Las poblaciones de chinches de cama en los Estados Unidos se han incrementado

sustancialmente en los últimos 15 años.[5,25] Mientras tanto, las estimaciones recientes sugieren que por lo menos 80,000[26] y posiblemente, hasta 300,000[27] residentes de los Estados Unidos en la actualidad están infectado con *T. cruzi*. En los Estados Unidos, la posibilidad de transmisión de *T. cruzi* mediante chinches de cama está, por lo tanto, a un nivel históricamente elevado.

Igualmente preocupante es la reciente aparición de nuevas poblaciones de chinches de cama en áreas endémicas de *T. cruzi* en América Latina. Este estudio fue motivado en parte por reportes, los cuales confirmamos, de poblaciones emergentes de *C. lectularius* en la ciudad de Arequipa, Perú, donde la enfermedad de Chagas sigue siendo un problema urbano.[16,28,29] Aunque el chinche de cama común y el tropical (*C. hemipterus*) han habitado ciertas áreas endémicas de Latinoamérica para la enfermedad de Chagas, han habido pocos esfuerzos para distinguir la transmisión de *T. cruzi* causada por chinches de cama de aquellas causadas por triatominos, tal vez porque previamente, ambos insectos podían ser eliminados a través de la aplicación de insecticidas piretroides en las viviendas. Las poblaciones de chinches de cama que han resurgido en los recientes años son altamente resistentes a los piretroides[30] y otras clases de insecticidas,[31] y el costo para eliminarlos de las casas está en un orden de magnitud mayor que el costo para el control de triatominos.[32,33] Si este resurgimiento de chinches de cama llega a ser prevalente en áreas de alta carga de la enfermedad de Chagas, ellos pueden suponer un serio desafío para la eliminación de la transmisión vectorial de la enfermedad de Chagas en el cono sur de Sudamérica y otras regiones.

Agradecimientos: Los autores agradecen a los siguientes miembros de la Estación de Campo de la Enfermedad de Chagas de la Universidad Peruana Cayetano Heredia en Arequipa, Perú, por sus útiles contribuciones a este proyecto: Carlos Condori, Gabriela Bustamante, Amparo Toledo, Javier Quintanilla, Jorge Apaza, Lina Mollesaca, Patricia Escalante, Oswaldo Cornejo, Roger Quispe, y María del Carmen Rojas. Los autores también agradecen a Dylan Tracy y Yage Wu por su ayuda con grabar en video el experimento del momento de la defecación. Además, los autores agradecen a Irving Nachamkin, Brian Strom, y Robert Gross por los comentarios sobre una versión anterior del manuscrito.

Apoyo financiero: Este trabajo fue financiado por la Universidad de Pennsylvania.
